# Work Ability After Anterior Cervical Decompression and Fusion Followed by a Structured Postoperative Rehabilitation: Secondary Outcomes of a Prospective Randomized Controlled Multi-Centre Trial with a 2-year Follow-up

**DOI:** 10.1007/s10926-021-10015-6

**Published:** 2021-12-11

**Authors:** Anneli Peolsson, Johanna Wibault, Håkan Löfgren, Åsa Dedering, Birgitta Öberg, Peter Zsigmond, Charlotte Wåhlin

**Affiliations:** 1 Department of Health, Medicine and Caring Sciences, Unit of Physiotherapy, Linköping University, Lasarettsgatan, House 511, 14th floor, 58183 Linköping, Sweden; 2 Occupational and Environmental Medicine Center, Department of Health, Medicine and Caring Sciences, Unit of Clinical Medicine, Linköping University, Linköping, Sweden; 3 Department of Activity and Health, and Department of Health, Medicine and Caring Sciences, Linköping University, Linköping, Sweden; 4 Neuro-Orthopedic Center, Jönköping, Region Jönköping County, and Department of Clinical and Experimental Medicine, Linköping University, Linköping, Sweden; 5 Department of Neurobiology, Care Sciences and Society, Division of Physiotherapy, Karolinska Institutet, Stockholm, Sweden; 6 Department of Neurosurgery, Linköping University Hospital, and Department of Clinical and Experimental Medicine, Linköping University, Linköping, Sweden; 7 Unit of Intervention and Implementation Research for Worker Health, Karolinska Institutet, Stockholm, Sweden

**Keywords:** Work ability, Spine, Cervical radiculopathy, Surgery, Rehabilitation

## Abstract

*Purpose* Information on work ability after ACDF and postoperative rehabilitation is lacking. The aim of the present study is therefore to investigate the work ability benefits of a structured postoperative treatment (SPT) over a standard care approach (SA) in patients who underwent anterior cervical decompression and fusion (ACDF) for cervical radiculopathy and factors important to the 2-year outcome. *Methods* Secondary outcome and prediction model of a prospective randomized controlled multi-centre study with a 2-year follow-up (clinicaltrials.gov NCT01547611). The Work Ability Index (WAI) and Work Ability Score (WAS) were measured at baseline and up to 2 years after ACDF in 154 patients of working age who underwent SPT or SA after surgery. Predictive factors for the WAI at 2 years were analysed. *Results* Both WAI and WAS significantly improved with SPT and SA (p < 0.001), without any between-group differences. Thoughts of being able to work within the next 6 months, Neck Disability Index (NDI), and work-related neck load explained 59% of the variance in WAI at the 2-year follow-up after ACDF. *Conclusions* Patients improved over time without group differences, suggesting the improvement to be surgery related. Expectation to work within the next 6 months, self-reported neck functioning and work-related neck load were important to work ability and are central factors to ask early after ACDF, to identifying further interventions promoting return to work.

## Introduction

The incidence of cervical radiculopathy (CR) has been reported to be 83.2 per 100 000 in the general population [1]. Slightly more men than women have been reported to have CR, and acute disc prolapse to be most common in the fourth and fifth decades of life [1, 2]. Patients with CR due to cervical disc disease (herniation and/or spondylotic changes) present with complex symptomatology and report, in addition to pain, physical and psychological disability, low health-related quality of life, and low work ability [3–5]. Decompression of the nerve root is the established surgical treatment for reducing pain and gaining neurological function, with an overall success rate of 80% on the modified Odom score (excellent to poor self-rated outcome) [3, 6]. However, disability (e.g. impairment in ventral and dorsal neck muscle endurance, neck range of motion, hand strength, balance, and self-reported neck-specific disability) and illness (i.e. low health-related quality of life) commonly remain [3, 4]. Therefore, evidence-based rehabilitation programmes are needed to improve patients’ function and health after intensive pain and the reduced functional ability and activity that precede cervical spine surgery. To date, neck-specific exercises are the single most evidence-based treatment in other types of neck pain disorders [7] and have been shown to be tolerated postoperatively by patients with CR without any harm [8, 9]. Neck-specific exercises with and without a behavioural approach [10], or a behavioural approach alone [11], have been shown to improve work ability in individuals with whiplash-associated disorders (WADs).

Work ability has been rarely studied in CR patients [5, 12–14] and, to the best of our knowledge, no study has used a validated work ability scale after surgery or after postsurgical rehabilitation for CR. As many patients affected by CR are middle-aged working population, work ability is an important outcome measure that needs to be studied. Knowledge of work ability in CR patients is scarce and needs to be investigated further in prospective studies with follow-up after anterior cervical decompression and fusion (ACDF). Increased knowledge may be helpful in increasing work ability in CR patients. The aim of this study was to investigate the outcome of work ability and the additional benefits of a structured postoperative treatment (SPT) including neck-specific exercise combined with a cognitive behavioural approach over a standard care approach (SA) at the 2-year follow-up in patients with MRI evidence of disc herniation and concomitant clinical signs who underwent ACDF for CR. A secondary aim was to investigate baseline (including surgery-related data) and short-term (3-month) factors important for improved work ability 2 years after ACDF.

This is the first prospective randomized controlled study (RCT) of postoperative rehabilitation consisting of neck-specific exercises combined with a behavioural approach in patients with CR.

## Materials and Methods

### Design

This is a report of the 2-year follow-up of work ability from the secondary analysis of a multi-centre RCT (ClinicalTrials.gov NCT01547611, March 8, 2012) performed according to a published study protocol [15]. The project leader and the investigator were blinded to group randomization and were not involved in either surgery or rehabilitation. The study was approved by the regional ethics committee in Linköping, Sweden, and was conducted according to the Declaration of Helsinki.

### Randomization

After obtaining informed consent, patients were pre-operatively randomized to SPT, which combined neck-specific exercise with a behavioural approach, or SA, in which patients were not referred to a physiotherapist after surgery [9, 15]. A computerized randomization list created by a statistician (before the study started) was used and handled by the central project leader, who was not involved in the treatment.

### Study Criteria for Participation

Patients were consecutively recruited from and underwent surgery at four neurosurgery/neuro-orthopaedic clinics in Sweden from 2009 to 2012. For a more homogenous population, only those who underwent ACDF were included in the present secondary analysis, excluding those who underwent a posterior surgery. The patients who underwent a posterior surgery were more often in a minority, older, male, and operated at several levels (p < 0.001) compared to ACDF patients and are commonly separated from ACDF in reports of outcomes. As the official retirement age in Sweden is 65 years and the present study included a 2-year follow-up regarding work ability, only individuals ≤ 63 years old at baseline were included in this report.

Criteria for inclusion in the present analysis of secondary outcomes in the RCT were as follows: surgery for cervical disc disease via ACDF at one to three segment levels because of radiculopathy, at least 2 months of persistent nerve root pain, clinical findings of nerve root compression compatible with verified cervical disc disease determined by magnetic resonance imaging, and age 18–63 years at baseline.

Exclusion criteria were myelopathy, previous fracture or luxation of the cervical column, malignancy or spinal tumour, spinal infection, previous surgery in the cervical column, systematic disease or trauma that contraindicated either the treatment programme or the measurements, diagnosis of a severe psychiatric disorder, such as schizophrenia or psychosis, known drug abuse, and lack of familiarity with the Swedish language (unable to understand and answer the questionnaires).

### Participants and Surgical Procedure

A total of 202 participants were randomized, 201 of whom (105 [52%] men) finally underwent surgery (mean age ± standard deviation: 50 ± 8.4 years) [9, 15]. Of the 201 patients included in the RCT, 43 did not fulfil the inclusion criteria for the present secondary analysis (38 because of a posterior surgery and 5 because of age > 63 years) and 4 did not answer the questionnaire regarding work ability at baseline. Thus, the present cohort consisted of 154 participants (Table 1). Based on the Standard for Swedish Occupational Classification (SSYK) [16], 25.8% were white collar workers (occupations with demands for a university education or similar, in professional, desk, managerial, or administrative work), 41.4% were pink collar workers (occupations without demands for a university education, in the care-oriented field, administrative work, or sales profession), and 32.8% were blue collar workers (skilled or unskilled manual labour; Table 1).

**Table 1:** Participant characteristics at baseline

	Total (n = 154)	SPT (n = 75)	SA (n = 79)	p-value
Gender, women, n (%)	85 (55.2)	44 (58.7)	41 (51.9)	0.399
Age, years, mean ± SD	48.0 ± 7.1	47.8 ± 7.3	48.2 ± 7.0	0.709
Smoking, yes, n (%)	38 (24.8)	17 (23.0)	21 (26.6)	0.606
Neck pain duration, n (%)				0.526
< 6 months	16 (11.8)	6 (9.5)	10 (13.7)	
6–12 months	49 (36.0)	21 (33.3)	28 (38.4)	
> 12 months	71 (52.2)	36 (57.1)	35 (47.9)	
Neck pain VAS, mm, mean ± SD	56.1 ± 23.8	56.1 ± 25.5	56.1 ± 22.3	0.990
Arm pain VAS, mm, mean ± SD	51.2 ± 28.5	56.2 ± 26.7	46.4 ± 29.4	0.034
NDI, %, mean ± SD	43.2 ± 14.6	42.8 ± 14.4	43.6 ± 14.9	0.767
Number of surgery levels, n (%)				0.204
1 level	95 (61.7)	51 (68.0)	44 (55.7)	
2 levels	57 (37.0)	23 (30.7)	34 (43.0)	
3 levels	2 (1.3)	1 (1.3)	1 (1.3)	
SSYK, n (%)				0.917
White collar	33 (25.8)	15 (25.0)	18 (26.5)	
Pink collar	53 (41.4)	26 (43.3)	27 (39.7)	
Blue collar	42 (32.8)	19 (31.7)	23 (33.8)	
Work situation, n (%)				0.577
Full-time work	81 (52.9)	36 (48.6)	45 (57.0)	
Part-time work	33 (21.6)	17 (23.0)	16 (20.3)	
No work	39 (25.5)	21 (28.4)	18 (22.8)	
Sick-leave, n (%)				0.922
No	96 (62.7)	46 (62.2)	50 (63.3)	
Part-time	36 (13.7)	11 (14.9)	10 (12.7)	
Full-time	21 (23.5)	17 (23.0)	19 (24.1)	
Work-related neck load, n (%)				0.550
Light	13 (8.9)	6 (8.5)	7 (9.3)	
Moderate	47 (32.2)	20 (28.2)	27 (36.0)	
Heavy	86 (58.9)	45 (63.4)	41 (54.7)	
Work within 6 months, n (%)				0.421
Very small chance	21 (14.7)	13 (18.6)	8 (11.0)	
Big chance	53 (37.1)	24 (34.3)	29 (39.7)	
Very big chance	69 (48.2)	33 (47.1)	36 (49.3)	
Changed work/work task because of the neck, no, n (%)	102 (70.8)	45 (67.2)	57 (74.0)	0.366
WAS, mean ± SD	3.8 (2.8)	3.7 (2.7)	3.8 (2.9)	0.932
WAI score, mean ± SD	28.8 ± 8.7	28.1 ± 8.7	29.4 ± 8.7	0.404
WAI score interval, n (%)				0.197
Poor	66 (45.8)	31 (46.3)	35 (45.4)	
Moderate	45 (31.3)	25 (37.3)	20 (26.0)	
Good	27 (18.8)	8 (11.9)	19 (24.7)	
Excellent	6 (4.2)	3 (4.5)	3 (3.9)	

Data are presented as n (%) or mean ± SD

*SPT* Structured Postoperative Treatment, *SA* Standard care Approach, *SD* Standard Deviation, *VAS* Visual Analogue Scale, *NDI* Neck Disability Index, *SSYK* Standard for Swedish Occupational Classification, *White collar workers* occupations with demands for a university education or similar, in professional, desk, managerial, or administrative work, *Pink collar workers* occupations without demands for a university education, in the care-oriented field, administrative work, or sales profession, *Blue collar workers* skilled or unskilled manual labour, *WAS* Work Ability Score, *WAI* Work Ability Index

Out of the 154 individuals, 115 (74.7%) had surgery at the C5-C6 level, 78 (50.6%) at C6-C7, 20 (135) at C4-C5, and 2 (1.3%) at C3-C4. Please observe that some individuals had multi-level surgery

The official retirement age in Sweden is 65 years. You can choose to retire earlier but receive a lower pension

Patients were operated on with ACDF using standard cages (i.e. filled with bone substitute or autologous bone collected during decompression; no iliac crest graft was taken). In most multilevel surgeries, an anterior plate was added to achieve primary stability.

### Description of the Post-Surgical Interventions

Patients in both groups: Patients in both groups received the same initial postoperative care at the surgical clinic during the first 6 weeks, which consisted of information, advice, and mobility exercises for the shoulders. After 6 weeks, patients were instructed to perform mobility exercises for the neck. Neither group used a cervical collar.

Structured postoperative treatment: Patients in the SPT group visited the physiotherapist (referral from the project team) once weekly beginning postoperative week 6, then twice weekly from postoperative week 12–24. Patients also performed exercises at home. SPT focused on facilitation and endurance of neck muscles, strengthening of scapular muscles, postural control, and increasing the overall physical activity level. Out of a standardized frame of exercises, the exercises were individually adjusted and progressed for each patient by the treating physiotherapist and registered in an exercise diary. A cognitive behavioural approach consisting of different lessons aimed at improving pain handling, coping strategies, ergonomics, and self-efficacy was included in the rehabilitation programme.

Standard care approach: Consistent with usual postoperative care after ACDF in Sweden, patients in the standard care approach group sought pragmatic postoperative physiotherapy themselves (61%), without the need for a referral, beginning postoperative week 6 if they felt it was needed.

### Main Outcome Measure

The Work Ability Index (WAI) [17] was used to assess the ability to work. The WAI is a reliable and valid self-report scale scored from 7 to 49, with higher scores indicating higher work ability [17]. Scores can be presented as mean values or categorized into four levels when used as an outcome measure: poor (7–27), moderate (28–36), good (37–43), and excellent (44–49) [18, 19].

The Work Ability Score (WAS) [20], also called the single-item WAI, as it is question number 1 in the WAI, was used in the analysis of between- and within-group differences at the 2-year follow-up. In the WAS, the present work ability is compared to life-time best. It is well validated and has a score of 0–10 points, with higher scores indicating higher work ability [20].

Both the WAI and WAS were measured at baseline, 1 year, and 2 years, and WAS also at the 6-month follow-up.

### The Prediction Model

The *dependent variable* was the WAI at the 2-year follow-up [17–19]. We investigated associations with background, baseline, and treatment-related data and 3-month follow-up data [21–30] included in the RCT [15].

After discussions between the researchers based on their theoretical knowledge of factors that may be important for work ability or CR patients, the following *independent variables* were included in the simple linear regression of the association with dependent variable Work Ability Index at the 2-year follow-up:

Background data; Age, gender, smoking, pain duration.

Baseline data; Intensity of neck pain, arm pain (Visual Analogue Scale (VAS), 0–100 mm, 0 = no pain, 100 = worst imaginable pain); Frequency of neck pain, arm pain, headache, numbness, hand weakness, being anxious (0 = never/daily, 1 = several times daily/always); Neck-specific function on the Neck Disability Index (NDI) (0–100%, 0% = no disability); Work-related neck load (1 = heavy, 2 = moderate, 3 = light); Thoughts of being able to work within the next 6 months (1 = very small chance/neither or, 2 = big chance, 3 = very big chance); Thrive on the work tasks; Expectation of surgery; Symptom satisfaction; Physical exercise level; VAS dizziness at rest; Distress and Risk Assessment Method (DRAM) (1 = distress: somatic or depression, 2 = at risk, 3 = normal); EuroQol five dimension (EQ-5D-3L) index (− 0.594 to 1, 1 = perfect health) and EQ-VAS (0–100, 0 = worst possible health, 100 = best possible health); Self-efficacy (0–200); CSQ (0–36 per subscale); Active range of motion of the neck in degrees; Hand grip strength in kilograms; Ventral and dorsal neck muscle endurance in seconds.

Treatment-related data; Number of surgery levels, randomization group.

Data at the 3-month follow-up after ACDF: Intensity of neck pain, arm pain, and headache now (VAS); Frequency of neck pain, arm pain, headache, numbness, hand weakness, being anxious; NDI; Thoughts of being able to work within the next 6 months; EQ-5D-3L index; EQ-VAS; Coping Strategy Questionnaire (CSQ).

### Statistical Analysis

The analysis was performed with an intention-to-treat approach. The overall baseline and follow-up characteristics between the two intervention groups were similar. Descriptive data are reported as mean ± standard deviation (SD) or as the number and proportion.

Linear mixed models (LMMs) with restricted maximum likelihood estimation and unstructured covariance structure were used to analyse between-group differences at each follow-up and between- and within-group effects over time on work ability based on the WAI (3 time points × 2 groups) and WAS (4 time points × 2 groups). LMMs can handle unbalanced data, which allows us to use all available data at each follow-up. Additional repeated contrast analysis comparing consecutive time points (baseline, 6 months, 1 year, and 2 years) was performed if the main effects or time-by-group interaction effects were significant. P < 0.05 was considered significant.

A simple linear regression analysis was used to analyse associations between independent variables (background, baseline, intervention factors and 3-month follow-up data) and the dependent variable WAI at the 2-year follow-up. P < 0.01 was considered to indicate a significant association to be further analysed in a final multiple linear regression model. If the independent variable appears at both baseline and the 3-month follow-up, the variable with the strongest association with the dependent variable were used in the multiple linear regression model. In all such cases, 3-month data had the strongest association. Multi-collinearity between x-variables was tested in a multiple model of all x-variables that significantly correlated with the y-variable in the univariate analysis. The variance inflation factor (VIF) was < 4, indicating that there were no problems with multi-collinearity.

The results from the simple linear regression models were presented as the coefficient of determination, R-squared (R^2^). The final multiple linear regression model was presented with an adjusted R^2^ and significant factors (p < 0.01) with beta coefficients, 95% confidence interval (CI) of beta, p-values, standardized beta coefficients, and partial eta squared (η_p_^2^) as a measure of the effect size (Table 2). The final model was based on 83 participants.

**Table 2:** The final multiple linear regression model for prediction of the Work Ability Index at 2 years

Variable	B	95% CI of B	p-value	β	η_p_^2^
Intercept	48.25	43.91 to 52.60	< 0.001		0.864
Work-related neck load			0.019		0.097
Heavy	− 6.00	− 10.85 to − 1.16	0.016	− 0.277	0.073
Moderate	− 2.13	− 7.17 to 2.90	0.401	− 0.091	0.009
Light (reference)					
Work within 6 months*			0.001		0.165
Very small chance	− 11.63	− 17.58 to − 5.67	< 0.001	− 0.393	0.164
Big chance	− 3.47	− 7.26 to 0.32	0.072	− 0.144	0.041
Very big chance (reference)					
NDI*	− 0.24	− 0.36 to − 0.11	< 0.001	− 0.363	0.152

*NDI* Neck Disability Index, *B* unstandardized beta coefficient, *CI* confidence interval, *β* standardized beta coefficient, *η*_*p*_^*2*^ partial eta squared

*Rated at 3-month follow-up

IBM SPSS Statistics for Windows version 26 was used for statistical analyses. All analyses were performed by a university statistician.

The required sample size was determined for the main outcome NDI [22]. The calculation was based on findings from a previous study [8] with an expected 10% between-group difference in the primary outcome of NDI (mean ± SD, 27 ± 18), assuming 80% power and a level of significance of 5%, with allowance for dropouts.

## Results

### Linear Mixed Models on WAI and WAS Over Time

**Fig. 1:**
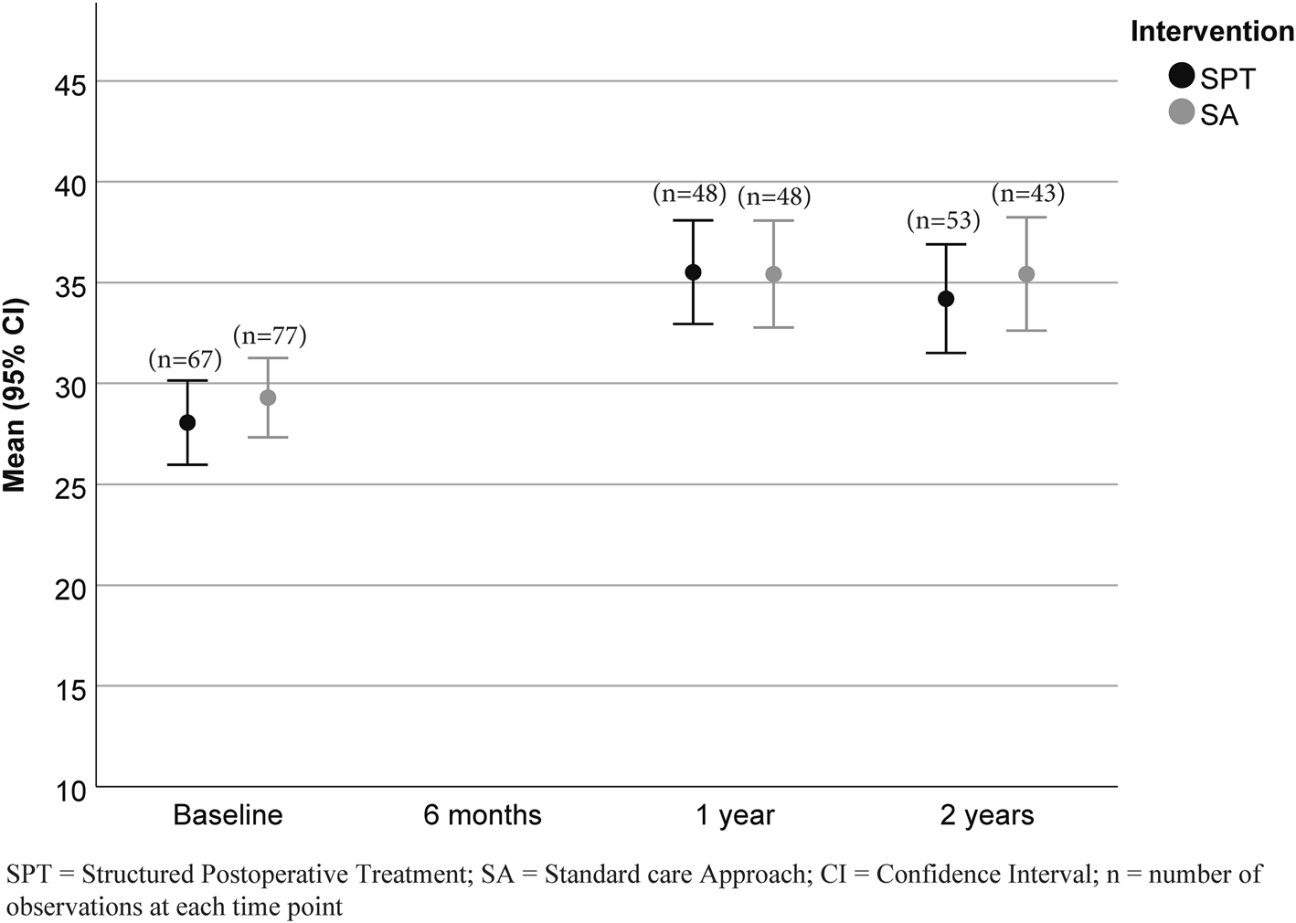
Work Ability Index at baseline and follow-up. The results are based on the total number of participants at each time point. *SA* standard care approach, *SPT* structured postoperative treatment

**Fig. 2:**
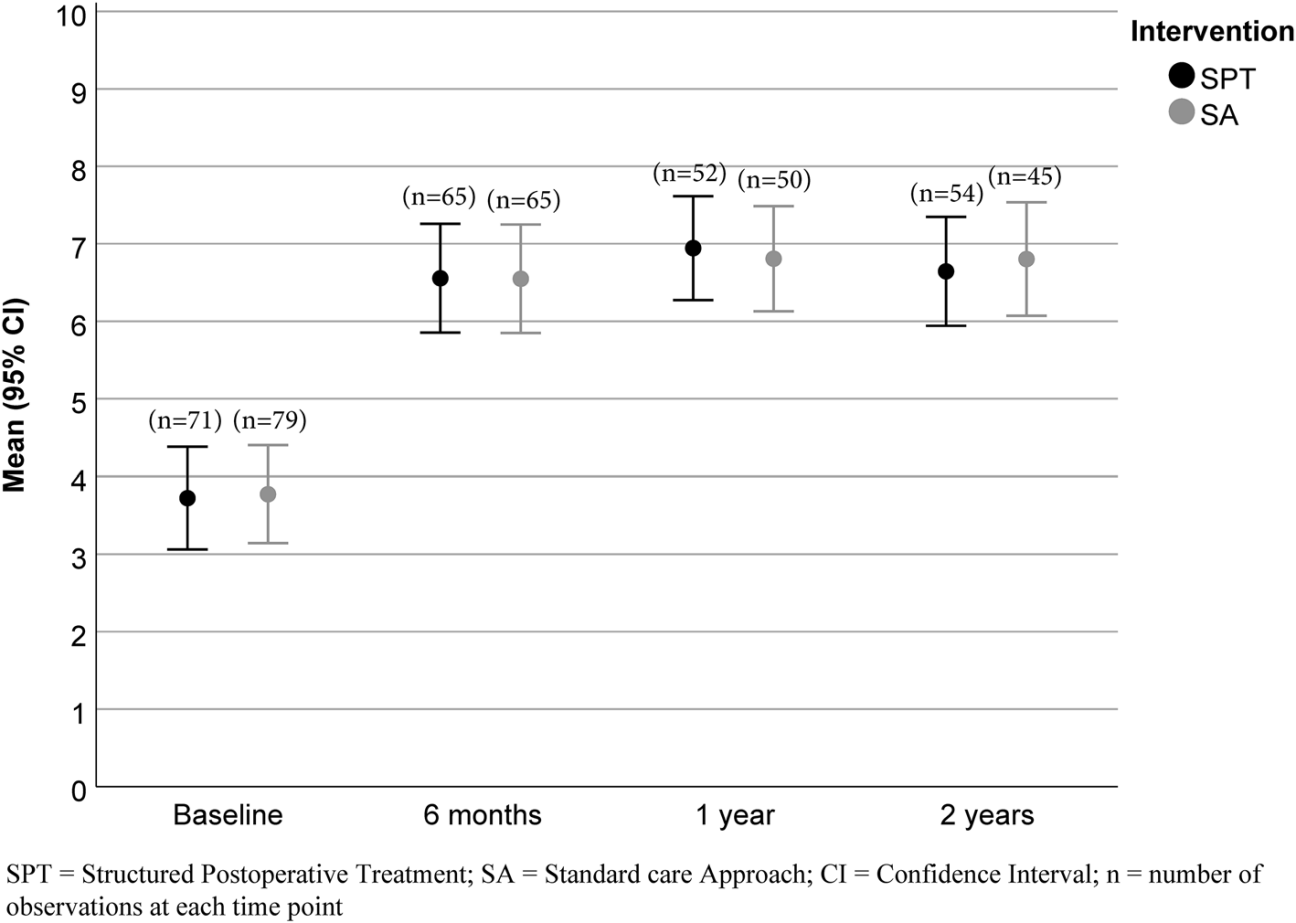
Work Ability Score at baseline and follow-up. The results are based on the total number of participants at each time point. *SA* standard care approach, *SPT* structured postoperative treatment

The LMM of WAI and WAS showed no significant main effect of group or the time-by-group interaction (WAI: group effect p = 0.606, time-by-group effect p = 0.466; WAS: group effect p = 0.966, time-by-group effect p = 0.852). However, the main effect of time was significant in both models (WAI, p < 0.001; WAS, p < 0.001), meaning that the WAI improved in both intervention groups (SPT: mean change 2.9, 95% CI 2.0–3.9, p < 0.001; SA: mean change 3.0, 95% CI 2.1–4.0, p < 0.001) and WAS (SPT: mean change 2.9, 95% CI 2.0–3.9, p < 0.001; SA: mean change 3.0, 95% CI 2.1–4.0, p < 0.001) from baseline to the 2-year follow-up (Figs. 1 and 2).

### Simple Linear Regression Models on WAI at the 2-year Follow-up

The following baseline variables were significantly (p < 0.01) associated with the WAI at the 2-year follow-up after ACDF: neck-related pain duration (R^2^ = 0.10), work-related neck load (R^2^ = 0.22), NDI (R^2^ = 0.11), Distress and Risk Assessment Method (DRAM) (R^2^ = 0.18) [25], self-efficacy (R^2^ = 0.09) [27], dorsal neck muscle endurance (R^2^ = 0.08) [21], and thoughts of being able to work within the next 6 months (R^2^ = 0.24). Significant variables at the 3-month follow-up were neck pain (R^2^ = 0.22), arm pain (R^2^ = 0.10), headache intensity now (R^2^ = 0.09) [18], neck pain frequency (R^2^ = 0.17), NDI (R^2^ = 0.42) [19], EQ-5D-3L index (R^2^ = 0.33), EQ-VAS (R^2^ = 0.29) [23], CSQ catastrophizing (R^2^ = 0.23) and pain behaviour (R^2^ = 0.08) [28], and thoughts of being able to work within the next 6 months (R^2^ = 0.40).

### Multiple Linear Regression Model on WAI at the 2-year Follow-up

The independent variables in the final multiple regression model explained 59% of the variance in the WAI at the 2-year follow-up after ACDF (p < 0.001). Three independent variables remained in the final model predicting high work ability on the WAI at the 2-year follow-up, in which both thoughts of being able to work within the next 6 months (η_p_^2^ = 0.165) and low NDI at the 3-month follow-up (η_p_^2^ = 0.152) had a large effect on the WAI, and low work-related neck load at baseline (η_p_^2^ = 0.097) had an intermediate effect on the WAI (Table 2).

## Discussion

There were no significant differences between the two rehabilitation groups, suggesting that there were no additional benefits of the structured physiotherapy intervention. In the present study, both randomization groups had a significantly improved WAI and WAS from baseline to the 2-year follow-up.

This finding is in line with the main outcome NDI, as well as pain variables, self-efficacy, and general health reported previously for the 2-year follow-up [9]. As there were no significant differences between SPT and SA, the results indicate that the main improvement at the 2-year follow-up is related to the surgical procedure.

Work ability is a broader concept than return to work or work capacity and has been defined e.g. in the work ability house model [31], including human resources, work arrangements, and management. Work ability can be seen as the balance between the person’s human resources and work and the demands, work arrangement, and management [31]. Half of those with WADs who return to work have reduced work ability [32]. A better health, work ability and positive expectations of return-to-work were associated with return-to-work for a working population with musculoskeletal disorders [33]. Work is important not only from an economic perspective, but also in regard to social roles, socialization, context, status, and general health [34, 35]. Work ability is a complex concept, and work environment and demands impact self-perceived work ability [31]. In the present study, 37.3% of the participants were on part- or full-time sick-leave before ACDF. As most of them have chronic pain (≥ 6 months), neck-specific disability, and a moderate or heavy work-related neck load, this may be considered to be low. However, this finding is in line with the results 10 years after ACDF in another Swedish report [3]. Being on sick-leave is dependent on the present system for sick leave in the country in question and the cultural context.

In individuals with chronic WADs, similar rehabilitation as in SPT has been shown to improve work ability based on the WAI and were significantly better than general physical activity [10]. Reviews regarding work-related neck pain have shown good results of strengthening exercises for the neck or increased physical activity in both neck pain and health-related quality of life [36, 37]. In the present study, SPT was tolerated without adverse events [9], and more studies regarding postoperative rehabilitation are needed to optimize care during the postoperative period, especially for those with remaining symptoms.

Thoughts of being able to work within the next 6 months as rated 3 months after ACDF was the strongest predictor, followed by NDI at 3 months and the baseline work-related neck load, which together explained 59% of the variance in the WAI at the 2-year follow-up and may be regarded as a rather strong model. Although baseline data have predictive value, short-term outcome data predict long-term follow-up to a greater extent. This has been reported for pain and disability after ACDF [38] and was not a surprise for the WAI.

The EQ-5D-3L index and pain scores are additional factors important for return to work as identified by Kim et al. [13] in retrospective studies of elective surgery for cervical degenerative disc disease. In a retrospective study of 67 consecutive patients, Bhandari et al. [12] reported that preoperative sick-leave, postoperative neck pain intensity, age, and disability were associated with return to work 1 year after anterior cervical discectomy. The differences between the studies may be explained by different study design, criteria, surgical method, and other variables in the prediction models. Cross-sectional analyses of associations between baseline variables and pre-surgery WAI from the same RCT [5] as the present study (but including patients who were retired and a few patients who underwent posterior neck surgery), identified self-efficacy, physical load on the neck at work, chance of being able to work within the next 6 months, coping strategies, hand weakness, and health-related quality of life to be related to WAI. Thus, physical load on the neck at work and chance of being able to work within 6 months are important factors associated with WAI at baseline, as well as predictive factors at the 2-year follow-up, showing continuity. In physiotherapy-treated individuals with CR due to degenerative disc disease in a study by Abbott et al. [14], NDI and the fear avoidance beliefs work subscale explained 65.8% of the variation in the WAI. Except for the cross-sectional baseline study by Ng et al. [5], disability has been an important factor in all of the studies mentioned above [12–14], as well as in the present study.

### Strengths and Weaknesses of the Study

Information about return to work is scarce for ACDF patients, and prospective randomized studies are lacking, as well as the use of a validated outcome measure. No previous study has investigated work ability after ACDF. The present study is the first prospective RCT investigating 2-year outcome and predictive factors regarding work ability. The weakness of the present study is that it was not designed for the WAI or WAS, but for the main outcome NDI [9]; therefore, individuals not of working age were included in the RCT, which reduces the number of individuals included in the present study.

### Implications for Practice

To in an early postoperative stage, asking patients about work tasks and physical load on their neck at work and their own thoughts of being able to work within the next 6 months will be reasonable in a clinical situation, as the WAI questionnaire may take too long time to fill in and analyse. Previous research on the working population [39] shows that ergonomic exposure at work with repetitive arm movement during most of the working day increase the risk of neck-shoulder pain. At the short-term follow-up after surgery, it is important to identify individuals who require further or extended interventions to gain and promote work ability [40]. A qualitative study on experiences with work ability in chronic WAD grade II and III showed that neck-specific exercise, information about the condition, and practical and emotional support from stakeholders need to be strengthened for increased work ability [41]. In a review of 18 qualitative studies [42], moderate evidence was found for collaboration between stakeholders; support from family, friends, colleagues, workplace settings, and health care personnel; and workplace adjustments and self-management strategies for return to work in individuals with musculoskeletal pain.

## Conclusion

Both rehabilitation groups significantly improved in work ability measured by the WAI and WAS, but without differences between SPT and SA, suggesting the improvement to be surgery related. Expectations of being able to work within the next 6 months was the strongest predictive factor, followed by NDI and work-related neck load, which constitute a rather strong model, explaining 59% of the variance in WAI at 2 years after ACDF. Simply asking patients at the short-term follow-up visit about their thoughts of being able to work within the next 6 months and to ask questions about work tasks and physical load on their neck at work is reasonable in the clinical setting. These are important question to be ask early after ACDF identifying those in need of further intervention to increase their work ability and supporting the process of returning to work after surgery.

## Data Availability

Data contain information regarding health and will not be available due to the Swedish health secrets act without a specific ethical permission.

## Code Availability

IBM SPSS version 26 was used for statistical analyses.

## References

[1] Radhakrishnan K, Litchy WJ, O’Fallon MV (1994). Epidemiology of cervical radiculopathy: A population-based study from Rochester, Minnesota, 1976 through 1990. Brain..

[2] Kelsey JL, Githens PB, Walter SD (1984). An epidemiological study of acute prolapsed cervical intervertebral disc. J Bone Joint Surg..

[3] Hermansen A, Hedlund R, Vavruch L (2011). A comparison between the carbon fiber cage and the Cloward procedure in cervical spine surgery. Spine..

[4] Hermansen AM, Cleland JA, Kammerlind AS (2014). Evaluation of physical function in individuals 11 to 14 years after anterior cervical decompression and fusion surgery–a comparison between patients and healthy reference samples and between 2 surgical techniques. J Manipulative Physiol Ther..

[5] Ng E, Johnston V, Wibault J (2015). Factors associated with work ability in patients undergoing surgery for cervical radiculopathy. Spine..

[6] Jacobs W, Willems PC, Kruyt M (2011). Techniques for single- and double-level cervical degenerative disc disease. Systematic review of anterior interbody fusion. Spine..

[7] Gross AR, Paquin JP, Dupont G (2016). Exercises for mechanical neck disorders: A Cochrane review update. Man Ther..

[8] Engquist M, Löfgren H, Öberg B (2013). Surgery versus nonsurgical treatment of cervical radiculopathy: a prospective, randomized study comparing surgery plus physiotherapy with physiotherapy alone with a 2-year follow-up. Spine..

[9] Peolsson A, Löfgren H, Dedering Å (2019). Postoperative structured rehabilitation in patients undergoing surgery for cervical radiculopathy: a 2-year follow-up of a randomized controlled trial. J Neurosurg Spine..

[10] Lo HK, Johnston V, Landén Ludvigsson M (2018). Factors associated with work ability following exercise interventions for people with chronic whiplash-associated disorders: secondary analysis of a randomized controlled trial. J Rehabil Med..

[11] Ehrenborg C, Gustafsson S, Archenholtz B (2014). Long-term effect in ADL after an interdisciplinary rehabilitation programme for WAD patients: A mixed-method study for deeper understanding of participants' programme experiences. Disabil Rehabil..

[12] Bhandari M, Louw D, Reddy K (1999). Predictors of return to work after anterior cervical discectomy. J Spinal Disord..

[13] Kim EJ, Chotai S, Wick JB (2019). Factors associated with return-to-work following cervical spine surgery in non-worker´s compensation setting. Spine..

[14] Abbott A, Allard M, Kierkegaard M (2020). What biopsychological factors are associated with work ability in conservatively managed patients with cervical radiculopathy?: a cross-sectional-analysis. PMR..

[15] Peolsson A, Öberg B, Wibault J (2014). Study protocol: Outcome of physiotherapy after surgery for cervical disc disease: a prospective randomised multi-centre trial. BMC Musculoskelet Disord..

[16] Wåhlin C, Ekberg K, Persson J, Bernfort L, Öberg B (2013). Evaluation of self-reported work ability and usefulness of interventions among sick-listed patients. J Occup Rehabil..

[17] Ilmarinen J (2007). The Work Ability Index (WAI). Occup Med..

[18] Smolander J, Blair P, Kohl H (2000). Work ability, physical activity, and cardiorespiratory fitness: 2-year results from project active. J Occup Environ Med..

[19] Pohjonen T, Ranta R (2001). Effects of worksite physical exercise intervention on physical fitness, perceived health status, and work ability among home care workers: five-year follow-up. Prev Med..

[20] El Fassi M, Bocquet Majery (2013). Work ability assessment in a worker population: comparison and determinants of work ability Index and work ability score. BMC Public Health..

[21] Briggs M, Closs JS (1999). A descriptive study of the use of visual analogue scales for the assessment of postoperative pain in orthopedic patients. J Pain Symptom Manage..

[22] Vernon H, Mior S (1991). The neck disability index: A study of reliability and validity. J Manipulative Physiol Ther..

[23] Cherkin DC, Deyo RA, Street JH (1996). Predicting poor outcomes of back pain seen in primary care using patients’ own criteria. Spine..

[24] Peolsson A, Almkvist C, Dahlberg C (2007). Age- and sex-specific reference values of a test of neck muscle endurance. J Manip Physiol Ther..

[25] Main CJ, Wood PLR, Hollis S (1992). The distress and risk assessment method: a simple patient classification to identify distress and evaluate the risk of poor outcome. Spine..

[26] Brooks R (1996). EuroQol: the current state of play. Health Policy..

[27] Altmaier E, Russell D, Feng KC (1993). Role of self-efficacy in rehabilitation outcome among chronic low back pain patients. J Couns Psychol..

[28] Rosenstiel AK, Keefe FJ (1983). The use of coping strategies in chronic low back pain patients: relationships to patient characteristics and current adjustment. Pain..

[29] Peolsson A, Hedlund R, Ertzgaard S (2000). Intra-and inter-tester reliability and age- and sex-specific range of motion of the neck. Physiother Can..

[30] Peolsson A, Hedlund R, Öberg B (2001). Intra- and inter-tester reliability and reference values for hand strength. J Rehabil Med..

[31] Ilmarinen J (2019). From work ability to research implementation. IJERPH..

[32] Kamper SJ, Rebbeck TJ, Maher CG (2008). Course and prognostic factors of whiplash: a systematic review and meta-analysis. Pain..

[33] Wåhlin C, Ekberg K, Persson J, Bernfort L, Öberg B (2012). Association between clinical and work-related interventions and return-to-work for patients with musculoskeletal or mental disorders. J Rehabil Med..

[34] Gebel M, Voβemer J (2014). The impact of employement transitions on health in Germany. A difference-in-difference propensity score matching approach. Soc Sci Med..

[35] Gnambs T, Stigbauer B, Selenko E (2015). Psychological effects of (non)employment: a cross-national comparison of the United States and Japan. Scand J Psychol..

[36] Varatharajan S, Côté P, Shearer HM (2014). Are work disability prevention interventions effective for the management of neck pain or upper extremity disorders? A systematic review by the Ontario protocol for traffic injury management (OPTIMa) collaboration. J Occup Rehabil..

[37] Louw S, Makwela S, Manas L (2017). Effectiveness of exercise in office workers with neck pain: a systematic review and meta-analysis. S Afr J Physiother..

[38] Peolsson A, Peolsson M (2008). Predictive factors for long-term outcome of anterior cervical decompression and fusion: a multivariate data analysis. Eur Spine J..

[39] Andersen LL, Vinstrup J, Sundstrup E, Skovlund SV, Villadsen E, Thorsen SV (2021). Combined ergonomic exposures and development of musculoskeletal pain in the general working population: A prospective cohort study. Scand J Work Environ Health..

[40] Adams H, Ellis T, Stanish WD, Sullivan MJL (2007). Psychosocial factors related to return to work following rehabilitation of whiplash injuries. J Occup Rehabil..

[41] Peolsson A, Hermansen A, Peterson G (2021). Return to work a bumpy road: A qualitative study on experiences of work ability and work situation in individuals with chronic whiplash-associated disorders. BMC Public Health..

[42] Liedberg GM, Björk M, Dragioti E (2021). Qualitative evidence from studies of interventions aimed at return to work and staying at work for persons with chronic musculoskeletal pain. J Clin Med..

